# The perceived and objectively measured effects of clinical pathways' implementation on medical care in China

**DOI:** 10.1371/journal.pone.0196776

**Published:** 2018-05-07

**Authors:** Jie Bai, Fei Bai, Hongbo Zhu, Di Xue

**Affiliations:** 1 Department of Hospital Management, School of Public Health, Key Laboratory of Health Technology Assessment, National Health Commission (Fudan University), Fudan University, Shanghai, P.R. China; 2 Center for Medical Service Administration, National Health Commission of China, Beijing, P.R. China; 3 Medical Administration and Management, Health and Family Planning Commission of Hubei Province, Wuhan, P.R. China; National Yang-Ming University, TAIWAN

## Abstract

**Introduction:**

Substantial resources have been expended on clinical pathways (CPs), but the reported effects of CPs on medical care vary considerably. This study sought to determine the effects of CPs on medical care in Chinese hospitals, including the perceived effects of CPs on medical care and the objectively measured patient outcomes.

**Methods:**

Study data were obtained from 54 public hospitals in three provinces of China in 2015. Hospital questionnaires, employee surveys, and chart reviews were used to collect data related to hospital characteristics, the implementation of CPs and compliance status, perceived effects of CPs, and objectively measured patient outcomes. Logistic regression models and linear regression models were adopted in this study.

**Results:**

The effects of CPs were not highly perceived by the hospitals or by the managers and physicians in China. The relatively low involvement in the implementation of and adherence to CPs resulted in CPs having no significant effects on hospital medical care as a whole. However, a chart review of 5 conditions in Chinese hospitals demonstrated that compliance with national CPs reduced the length of stay (LOS) and inpatient medical costs.

**Conclusions:**

CPs should be implemented widely and followed closely to improve hospital medical care as a whole, and further studies should be conducted to identify the key elements of the effects of CPs on patient clinical outcomes.

## Introduction

In times of substantial health system reforms, health care providers face new challenges, such as the introduction of a diagnosis-related group system, patients’ freedom of choice, demands to maintain high standards of care and to ensure that patients are as satisfied as possible, and volatile labour markets in health care [1]. To respond to these global trends, substantial resources have been expended on the development, implementation, and maintenance of clinical pathways (CPs) [2].

### Concepts of CPs

CPs are structured multidisciplinary care plans used by health services to detail essential steps in the care of patients with a specific clinical problem [2]. CPs can contribute to increased adherence to clinical guidelines (CGs), improved quality of care, decreased length of stay (LOS), and reduced hospital costs [3, 4]. As CPs are adopted worldwide, the implementation of national CPs is facilitated in health care reform by the National Health Commission (NHC, previously called the “Ministry of Health”) of China to improve the effectiveness and efficiency of medical care and to meet patient demands in hospitals [5].

### Implementation of and adherence to CPs

The implementation of CPs varies among different countries. At the beginning of this century, more than 80% of the hospitals in the United States were already using CPs for at least some of their interventions [6]. In China, 94.4% of the public hospitals had implemented CPs in 2015. Among those that implemented CPs, an average of 45 CPs were implemented, and an average of 52.7% of cases adopted a CP [7]. Furthermore, less than 75% of physicians have implemented CPs as of 2015 [8]. Adherence to CPs also differs among different countries and different conditions [5,9–11]. Since CPs cannot be effective if they are not applied [3], many researchers analyse the factors that affect the implementation of and adherence to CPs [4,12–14]. Two reasons that physicians or other providers do not apply CPs are lack of proper attitudes and lack of motivation [4,12,15]. Therefore, the benefits of implementing CPs should be demonstrated to ensure that physicians and other providers develop proper attitudes towards evidence-based CPs and therefore motivate them to follow CPs.

### Effects of CPs on medical care

A systematic review of 27 studies showed that stand-alone CPs reduced LOS in most studies, that hospital costs/charges and in-hospital complications also decreased, and that documentation improved. However, there was no evidence of differences in hospital readmission or in-hospital mortality between the stand-alone CP group and the usual care group [2]. A study that evaluated the effects of implementation of a CP into routine practice for breast surgery found that the CP decreased LOS by 24% and the total cost per case by more than 13% with no increase in the number of readmissions [16]. The implementation of a fast-track CP led to reduced postoperative LOS and hospital charges for high-risk patients undergoing elective liver resection with no differences in complications, mortality, or readmission rate [17]. A systematic review of 7 randomized controlled trials and controlled clinical trials published from 1980 to 2013 demonstrated that the application of a CP for laparoscopic cholecystectomy effectively reduced hospital LOS and total costs, but there was insufficient evidence to demonstrate a reduction in postoperative complications [18]. A self-paired comparison of perioperative outcomes in patients undergoing total knee arthroplasty demonstrated that the use of a CP reduced LOS and improved clinical outcomes and patient satisfaction while reducing costs for identical surgical procedures [19]. A literature review of the effect of CPs on the in-hospital management of COPD showed that there were positive effects on blood sampling, daily weight measurements, arterial blood gas measurements, referral to rehabilitation, feelings of anxiety, LOS, readmission, and in-hospital mortality [20]. The utilization of a CP in mental health services greatly supported both the documentation of clinical processes and fidelity to CGs in early psychosis treatment, and excellent adherence (over 80%) led to a reduction in client trauma [21]. A retrospective study of consecutive cytoreductions showed that the failure-to-rescue rate significantly decreased and the quality of care improved after the introduction of a CP [22]. A study in Australian hospitals reported that a CP for chronic cough in children improved clinical outcomes [23]. However, the results of studies regarding the effects of CPs on medical care vary considerably [2].

### Indicators and outcomes used to evaluate the effects of CPs

To measure the effects of CPs, intermediate and discharge outcomes must be appropriately defined and quantified, and the critical indicators for achieving each outcome must be measured incrementally such that diagnostic variance data can be obtained and analysed [24]. Five domains of the Leuven Clinical Pathway Compass were suggested to measure the effects of CPs, including the “clinical domain”, “service domain”, “team domain”, “process domain”, and “financial domain” [25]. In empirical studies, LOS, hospital cost/charges, and complications are frequently used to measure the cost-effectiveness of CPs, whereas hospital readmissions, health-related functioning, quality of life, health status, documentation, patient satisfaction, work satisfaction and other indicators are used less frequently [26].

The aim of this study was to determine the effects of CPs on medical care in the hospital setting, including the perceived effects of CPs on medical care and objectively measured patient outcomes.

## Materials and methods

### Survey samples

This study was conducted in Shanghai, Hubei Province and Gansu Province to represent high, middle, and low levels of socioeconomic status and eastern, central, and western territorial locations of China.

In Hubei and Gansu Provinces, 3 areas (cities or autonomous prefectures) were selected to represent the high, middle, and low levels of socioeconomic status in the province. In each surveyed area of Hubei and Gansu Provinces, 2 tertiary and 4 secondary general public hospitals were chosen as the surveyed hospitals. Among these hospitals, 1 tertiary and 1 secondary general public hospital were chosen from each surveyed area as the hospitals in which chart reviews were conducted.

Because the tertiary general public hospitals in Shanghai were not evenly distributed among districts and there were not enough secondary general public hospitals in each district, 6 tertiary general public hospitals were selected in Shanghai to represent the tertiary hospitals owned by universities, by Shanghai governments, or by district governments, and 12 secondary general public hospitals were selected from 4 districts of Shanghai to represent the hospitals in urban, suburban, and rural areas. Among these hospitals, 3 tertiary general public hospitals and 3 secondary general public hospitals were selected for chart reviews to represent different types of ownership of tertiary general public hospitals and to represent hospitals in different types of areas.

### Data sources

#### Hospital survey

Fifty-four hospitals in three provinces (including 18 tertiary general public hospitals and 36 secondary general public hospitals) were surveyed using questionnaires. The paper-based hospital questionnaires were completed by managers in the medical affairs office and other relevant management offices in the surveyed hospitals. The data collected by the survey related to hospital characteristics, the number of CPs that each hospital implemented, and patient outcomes that are routinely reported to the government (cured or improved rate at inpatient discharge, LOS, and average inpatient medical cost). A hospital-level assessment of the effects of CPs on medical care was also included in the survey. According to the Leuven Clinical Pathway Compass, which includes the “clinical”, “service”, “team”, “process” and “financial” domains [25], the items for the hospital-level assessment in the study also included those from the “clinical domain” (medical effectiveness, patient safety, and readmission of inpatients within 30 days), “service domain” (patient satisfaction), “team domain” (employee satisfaction), “process domain” (standard medical care, variations in medical care, appropriate use of antibiotics, and quality of documentation), and “financial domain” (average LOS and medical cost). These items were assessed using a Likert scale (scores 1–5), with 1 indicating “strongly disagree” and 5 indicating “strongly agree”. To clarify the overall assessment of the CPs’ effects on medical care, we added an item, “the overall positive effects on medical care”, to the survey.

#### Employee survey

Surveys of employees in each of the 54 sampled hospitals were conducted using paper-based questionnaires. Ten percent of managers (at least 15 managers) and 10% of physicians (at least 15 physicians) were randomly selected to participate in the survey. In this study, “manager” refers to employees with management responsibilities at high or middle levels, not including physician-managers, nurse-managers, and technician-managers. The rank scale for assessing the perceived effects of CPs in the employee survey was the same as that in the hospital survey. The employee survey also collected data about employee characteristics, whether employees personally implemented CPs, and the percentage of inpatients for whom they implemented CPs.

#### Chart review

Five conditions were selected for chart reviews: community-acquired pneumonia (“pneumonia”), acute myocardial infarction (AMI), acute left ventricular failure (“heart failure”), planned caesarean section (“C-section”), and gallstones associated with acute cholecystitis and undergoing cholecystectomy (“cholecystectomy”). These 5 conditions covered medical care in internal medicine, surgery, and obstetrics, were common in general hospitals, had national CPs published by the NHFPC, and had CPs/guidelines in both China and at least one developed country.

We identified all the patients with a given diagnosis, based on inpatient international classification of disease (ICD-10 or ICD-9) codes, who were admitted to each hospital during 2014 for each condition. In most hospitals, hospital information systems were used to identify the patients and collect the first page of their medical records, including patient characteristics, diagnoses, clinical outcomes (cured, improved, died, or other), LOS, and medical costs. However, for hospitals in rural areas that had no electronic health information system, patient identification and collection of the first page of the medical records were conducted manually.

To ensure that the sample was evenly distributed throughout the year, we selected 2–3 cases for each condition from each month so that 30 cases for each condition in one hospital were sampled. If a hospital admitted fewer than 30 patients for a particular condition in 2014, all the medical records in 2014 and some medical records from late 2013 for this condition were extracted for this hospital to obtain 30 records.

We developed an audit chart with key process indicators (KPIs) for each of the 5 conditions, and each KPI was followed by relevant information items that needed to be completed by auditors when viewing patient medical records. The KPIs were generated based on the CPs published by the NHFPC, focusing on indicators that were important determinants of quality of care, such as timely examinations, adequate medication, reasonable treatment, and LOS, and that were likely to be available in the medical records (see S1–S5 Tables).

The auditors then extracted information from the medical records corresponding to each item in the audit chart for each patient. To ensure the quality and consistency of the chart audits, we trained eighteen auditors (master’s or PhD students with specialties in social medicine and health service management) on the meaning of each item on the checklist and how to judge whether the KPI was achieved. In addition, ten experts were invited to review the medical records of 5 conditions in two hospitals; the auditors reviewed the same medical records to analyse the consistency of medical reviews between experts and auditors. One inspector was also assigned to check 10% of the reviewed charts for each condition in each hospital. The consistency rates between the auditors and experts and between the auditors and inspectors were 90.82% (kappa = 0.7286, P<0.001; McNemar χ^2^ = 15.21, P<0.0001) and 90.88% (kappa = 0.7489, P<0.001; McNemar χ^2^ = 2.67, P = 0.1025), respectively.

### Data analyses and quality control

We analysed the implementation rates of CPs at the hospital and physician levels in 54 hospitals. We also analysed the average number of CPs that were applied in 51 hospitals that adopted at least one CP.

When we analysed the rates of compliance with national CPs for the 5 conditions in 18 hospitals, we defined physicians as compliant for an indicator only if the care was consistent with the requirement of the CP, and the information was recorded in the medical record or if the medical record included a reasonable explanation for the lack of compliance. For each particular KPI, the auditors judged whether the patient received fully compliant care and then coded the result as “1” or “0”. We then calculated the proportion of KPIs that were met for each individual patient for each condition. This patient-level measure was used to calculate the average of the proportion of KPIs that were met across all the patients with a given condition (average compliance rate).

In the study, t tests were used to compare the hospital-level assessment of the CPs’ effects on medical care against individual perceptions and to compare managers’ perceptions against physicians’ perceptions.

Logistic models were adopted to analyse whether the physicians who implemented the CPs had a more favourable perception of the CPs’ effects on medical effectiveness, LOS and inpatient cost while controlling for hospital location and level as well as physician characteristics. In these models, if the rank scores assessed by a physician were greater than the corresponding median scores of all the physicians, then the dependent variables were coded as “1”; otherwise, they were coded as “0”. Logistic models were also adopted to analyse whether the number of implemented CPs in hospitals affected objectively measured patient outcomes at the hospital level (including actual cured or improved rate, average LOS, and average inpatient cost). In the logistic models, if the original dependent variables were greater than their corresponding median, then the dependent variables in the models would be equal to 1; otherwise, they would be equal to 0.

Logistic models and linear regression models were applied to analyse whether the compliance rate affected objectively measured patient outcomes for each of the 5 conditions while controlling for hospital fixed effects. Logistic models were applied to analyse the CPs’ effects on the actual effectiveness of medical care, in which the dependent variable would be equal to 2, 1 or 0 for cured status, improved status or other status at patient discharge, respectively. Linear regression models were applied to analyse the CPs’ effects on actual patient LOS and inpatient cost. In the linear regression models that analysed the effects of compliance rates on LOS, the average compliance rates of KPIs were calculated without the KPI of LOS because of the high correlation. In the linear regression models that analysed the effects of compliance rates on inpatient cost, the dependent variable (inpatient cost) was log10-transformed.

This study was approved by the Institutional Review Board (IRB) of the School of Public Health, Fudan University (IRB #2014-03-0502). The IRB waived informed consent for the chart review and written informed consent for the employee survey in the hospitals.

## Results

In this study, all 54 hospitals in 10 areas of Shanghai, Hubei Province and Gansu Province responded to our questionnaire survey, and 1,638 physicians and 316 managers in the surveyed hospitals returned the questionnaires. In addition, chart reviews of 534 cases of pneumonia, 487 cases of AMI, 426 cases of heart failure, 538 cases of C-section, and 536 cases of cholecystectomy were conducted.

### Implementation and compliance rates of CPs

This study showed that the number of CPs applied in the surveyed hospitals in China ranged from 4 to 128. Furthermore, 71.84% of the responding physicians implemented CPs, but 32.84% of them implemented CPs on less than 20% of their inpatients.

Among the 70 most common conditions for which physicians implemented CPs, pneumonia, AMI, and C-section were listed among the top four (accounting for 7.25%, 7.07%, and 5.43%, respectively), whereas gallbladder stones with chronic cholecystitis and heart failure accounted for 3.62% and 1.81%, respectively, placing them among the top twenty conditions.

The review of medical charts showed that the average compliance rates for pneumonia, AMI, heart failure, C-section, and cholecystectomy were 65.07%, 68.87%, 68.04%, 77.36%, and 67.65%, respectively.

### Perceived effects of CPs on medical care

This study found that the level of overall positive effects of CPs on medical care was perceived as moderate by both the surveyed hospitals (average score = 3.57) and the surveyed individuals (managers and physicians) in the hospitals (average score = 3.66) (Table 1).

**Table 1:** Perceived effects of CPs on medical care (at the hospital and individual levels) ^†^.

	Hospital level		Individual level				Individual level					
							Managers		Physicians			
Effects	Mean	Std ^‡^	Mean	Std ^‡^	t value		Mean	Std ^‡^	Mean	Std ^‡^	t value	
Increase -in medical effectiveness	3.69	0.71	3.66	0.89	0.24		3.99	0.71	3.60	0.91	8.58	***
Increase in patient safety	3.78	0.70	3.70	0.87	0.69		4.04	0.69	3.63	0.89	9.07	***
Reduction in readmission of inpatients within 30 days	3.35	0.74	3.55	0.86	-1.61		3.82	0.78	3.50	0.87	6.65	***
Increase in patient satisfaction	3.57	0.85	3.59	0.90	-0.17		3.87	0.78	3.54	0.92	6.75	***
Increase in standard medical care	3.92	0.66	3.78	0.82	1.46		4.06	0.67	3.73	0.84	7.61	***
Reduction in variations in medical care	3.88	0.65	3.67	0.87	2.23	*	4.00	0.70	3.61	0.88	8.64	***
Facilitation of appropriate use of antibiotics	3.88	0.59	3.82	0.78	0.72		4.08	0.68	3.77	0.78	7.19	***
Improvement in documentation	3.69	0.71	3.79	0.82	-1.01		4.05	0.69	3.74	0.83	7.22	***
Increase in employee satisfaction	3.29	0.81	3.44	0.93	-1.11		3.74	0.82	3.38	0.94	6.92	***
Reduction in average LOS	3.82	0.62	3.70	0.88	1.35		4.03	0.68	3.64	0.90	8.87	***
Reduction in medical cost	3.49	0.70	3.61	0.87	-0.99		3.88	0.75	3.56	0.88	6.68	***
Overall positive effects on medical care	3.57	0.64	3.66	0.86	-1.03		3.95	0.70	3.61	0.88	7.57	***

† Full score = 5

‡ standard deviation

*** P<0.001.

The level of positive effects of CPs on standard medical care, variations in medical care, use of antibiotics, quality of documentation, and average LOS was perceived as relatively higher at the hospital and individual levels (scores ranged from 3.69 to 3.92 and from 3.67 to 3.82, respectively) than that on other aspects. However, the positive effects of CPs on medical effectiveness, readmission of inpatients within 30 days, patient satisfaction, and medical costs were perceived as lower at the hospital and individual levels (scores ranged from 3.35 to 3.69 and from 3.55 to 3.66, respectively). At the hospital and individual levels, CPs were perceived to have the lowest positive effects on employee satisfaction (scores of 3.29 and 3.44, respectively) (Table 1).

The survey found no significant difference in the perception of the effects of CPs between the hospital level and the individual level, except that a higher effect of CPs on reducing variation was perceived at the hospital level than at the individual level (3.88 vs. 3.67) (Table 1).

Furthermore, this study determined that positive perceptions of the CPs’ effects on all the surveyed aspects were lower among the physicians in the hospitals than those among the managers, particularly regarding medical effectiveness (3.60 vs. 3.99), patient safety (3.63 vs. 4.04), variations in medical care (3.61 vs. 4.00), and average LOS (3.64 vs. 4.03) (Table 1).

### Influence of the implementation of CPs on physicians’ perceptions

After using logistic models to control for hospital location and level and for physician characteristics (gender, age, and education), this study did not show a higher positive perception of the effects of CPs for the physicians who implemented CPs (Table 2).

**Table 2:** Logistic models for physician-perceived effects of CPs on medical care^†^.

Parameters	Increased medical effectiveness			Reduced LOS				Reduced inpatient cost			
	β	SE	χ^2^_Wald_	β	SE	χ^2^_Wald_		β	SE	χ^2^_Wald_	
Intercept	0.28	0.30	0.86	0.32	0.31	1.06		0.04	0.30	0.02	
Shanghai	0.10	0.11	0.74	0.02	0.11	0.03		0.02	0.11	0.05	
Gansu Province	0.25	0.14	3.11	0.56	0.15	14.07	***	0.46	0.14	10.65	**
Tertiary hospitals	0.15	0.11	1.85	0.07	0.11	0.41		0.05	0.11	0.26	
Physicians who implemented CPs (1: yes, 0: no)	-0.11	0.10	1.30	0.07	0.10	0.43		0.09	0.10	0.87	
χ^2^ likelihood		8.48			22.69	**			21.32	**	

† Logistic models were used, controlling for physician characteristics (gender, age, and education). If the original dependent variables were greater than their corresponding median, then the dependent variables in the model were coded as “1”; otherwise, they were coded as “0”.

** P<0.01

*** P<0.001

### Objectively measured effects of CP implementation

This study did not determine that the number of implemented CPs affected patient outcomes (actually cured or improved rate at discharge, average LOS, and average inpatient medical cost) at the hospital level after controlling for hospital location and level (Table 3).

**Table 3:** Logistic models for the objectively measured effects of CPs at the hospital level^†^.

	Cured or improved rate (%)			Average LOS			Average inpatient cost			
Parameters	β	SE	χ^2^_Wald_	β	SE	χ^2^_Wald_	β	SE	χ^2^_Wald_	
Intercept	-0.30	0.67	0.21	0.38	0.67	0.32	-1.33	0.93	2.04	
Shanghai	-1.29	0.75	2.97	-0.73	0.70	1.06	4.00	1.25	10.19	**
Gansu Province	1.05	0.72	2.12	-1.20	0.72	2.75	-0.91	0.97	0.88	
Tertiary hospitals	-0.66	0.77	0.73	0.94	0.74	1.63	3.41	1.13	9.11	**
No. of implemented CPs	0.01	0.01	0.89	0.001	0.01	0.004	-0.01	0.02	0.17	
χ^2^_likelihood_		10.57	*		5.41			37.06	***	

† Logistic models were used for the effects of CPs on medical care at the hospital level. If the original dependent variables were greater than their corresponding median, then the dependent variables in the model were coded as “1”; otherwise, they were coded as “0”.

* P<0.05

** P<0.01

*** P<0.001

The chart review found that the cases with higher compliance rates with national CPs had lower LOS and lower inpatient medical costs for all 5 conditions. However, there were mixed results on the effectiveness of medical care (cured, improved or other status of inpatients at discharge) among the 5 conditions (positive effect in pneumonia cases, negative effect in AMI cases, and no significant effect in heart failure, C-section and cholecystectomy cases) after controlling for hospital fixed effects and patient characteristics (Table 4).

**Table 4:** Multivariate models for the objectively measured effects of CPs at the case level^†^.

		Effectiveness ^‡^			LOS^§^			Inpatient cost^§^^※^		
	Parameters	β	χ^2^_Wald_		β	t value		β	t value	
Pneumonia										
	Intercept 2	-8.422	26.62	***	-	-		-	-	
	Intercept 1	-1.702	1.25		11.462	6.56	***	3.411	31.16	***
	Sex (1: male, 0: female)	-0.130	0.18		1.147	3.31	***	0.054	2.93	**
	Age (years)	-0.005	0.29		0.008	0.89		0.002	4.85	***
	Medical insurance (1: yes, 0: no)	0.127	0.01		0.939	0.87		0.067	0.87	
	Compliance rate^#^	4.380	7.45	**	-6.194	-3.43	***	-0.267	-2.77	**
	Model fit	479.94	***		4.23	***		23.39	***	
AMI										
	Intercept 2	-0.920	0.61		-	-		-	-	
	Intercept 1	3.878	10.68	**	14.407	4.72	***	4.099	25.64	***
	Sex (1: male, 0: female)	-0.077	0.08		-0.892	-1.25		0.045	1.18	
	Age (years)	-0.014	2.02		0.055	2.13	*	-0.002	-1.06	
	Medical insurance (1: yes, 0: no)	0.268	0.74		0.646	0.77		0.100	2.15	*
	Compliance rate^#^	-3.071	11.61	***	-14.186	-6.28	***	-0.805	-6.77	***
	Model fit	345.38	***		5.07	***		32.78	***	
Heart failure										
	Intercept 2	-2.342	4.16	*	-	-		-	-	
	Intercept 1	2.339	4.20	*	15.405	3.55	***	3.590	23.3	***
	Sex (1: male, 0: female)	0.069	0.08		-0.095	-0.10		-0.026	-0.76	
	Age (years)	-0.003	0.08		0.049	1.37		0.001	0.82	
	Medical insurance (1: yes, 0: no)	0.024	0.004		2.617	1.78		0.163	3.12	**
	Compliance rate^#^	-0.484	0.21		-16.393	-4.11	***	-0.352	-2.46	*
	Model fit	224.92	***		5.51	***		14.45	***	
C-Section										
	Intercept 2	3.198	3.09		-	-		-	-	
	Intercept 1	4.072	4.95	*	11.535	1.15	***	3.600	67.99	***
	Age (years)	-0.030	0.47		0.047	0.03		0.004	3.04	**
	Medical insurance (1: yes, 0: no)	-0.396	0.69		-0.334	0.27		-0.053	-4.18	***
	Compliance rate^#^	-1.392	0.41		-11.071	1.42	***	-0.175	-2.66	**
	Model fit	323.37	***		5.32	***		92.29	***	
Cholecystectomy										
	Intercept 2	-4.113	2.26		-	-		-	-	
	Intercept 1	0.337	0.02		26.471	10.8	***	4.138	56.88	***
	Sex (1: male, 0: female)	0.596	0.94		-0.787	-1.76		-0.004	-0.34	
	Age (years)	0.023	1.37		0.038	2.31	*	0.001	2.58	*
	Medical insurance (1: yes, 0: no)	0.729	0.70		0.688	1.07		0.026	1.29	
	Compliance rate^#^	6.130	3.54		-27.141	-10.41	***	-0.574	-7.42	***
	Model fit	388.21	***		14.38	***		60.13	***	

† Hospitals were controlled as fixed effects

‡ logistic models were used, and model fitness was tested using likelihood χ^2^

§ linear regression models were used, and model fitness was tested by the F value

※the dependent variable (inpatient cost) was log10-transformed in the models

# percentage of KPIs that complied with national CPs

* P<0.05

** P<0.01

*** P<0.001

## Discussion

### Relatively low perception of the effects of CPs

CPs have been designed as an approach that can be used to face the challenges in health care, ensuring an equally high standard of care, a foreseeable LOS, a minimized expenditure per treated case, and high patient and work satisfaction [1,3,4]. However, our study found that the overall positive effects of CPs on medical care were not highly perceived by the hospitals or by the managers and physicians in China (average scores of 3.57 and 3.66, respectively). The relatively low perception of the positive effects of CPs on medical care, particularly on medical effectiveness, readmission of inpatients within 30 days, patient satisfaction, and medical cost, may negatively affect the attitudes of hospitals and their managers and physicians towards CPs and therefore may affect their motivation to implement CPs. In addition, these relatively low perceptions may partly result in the relatively low involvement in the implementation of and adherence to CPs in Chinese hospitals. Of the average of 45 CPs that were applied in hospitals [7], only 72% of physicians implemented the CPs, and 33% of those physicians did so for less than 20% of their inpatients. The average compliance rates ranged from 65% to 78% for the 5 conditions.

Because medical care in hospitals is primarily led by physicians, their attitudes and motivation towards CPs are crucial for hospitals to apply CPs. However, our study revealed that the physicians had lower perceptions of the effects of CPs on medical care than the managers in Chinese hospitals, particularly regarding medical effectiveness, patient safety, variations in medical care, and average LOS (3.60–3.64 vs. 3.99–4.04, respectively). More studies are necessary to demonstrate the objectively measured effects of CPs on medical care (particularly on patient outcomes) to motivate physicians to implement and adhere to CPs.

### No objective effects of CPs on patient outcomes in hospitals as a whole

No objectively measured effect of CPs on patient outcomes was observed in the hospitals in this study. The number of implemented CPs in the hospitals did not affect the cured or improved rate at discharge, average LOS, or average inpatient medical cost in Chinese hospitals as a whole. One possible reason for these findings is the relatively low involvement in the implementation of and adherence to CPs in Chinese hospitals, which limited the effects of CPs on overall patient outcomes in the hospitals. The best CPs in the world will not improve patient outcomes if they are not put into practice and widely followed [15,27]. Other possible reasons include confounding factors that affect the validity of the outcome measures [26].

### Positive effects of CP compliance on LOS and medical costs at the case level

Most studies have demonstrated that implementing CPs can reduce LOS and medical costs, whereas some have found conflicting effects on medical care [2,3,16,17,20]. Our study also showed that cases with higher CP compliance rates had lower LOS and lower impatient medical costs for all 5 conditions after controlling for hospital fixed effects and patient characteristics. However, the strong positive effects of CPs on LOS and inpatient medical costs did not significantly enhance their positive perception by the physicians who implement the CPs.

In addition, our study found conflicting effects of compliance rates with national CPs on inpatient status at discharge among 5 conditions at the case level (positive effect in pneumonia cases, negative effect in AMI cases, and no significant effect in heart failure, C-section and cholecystectomy cases) after controlling for hospital fixed effects and patient characteristics. One possible explanation is that different characteristics of the different conditions respond differently to their CPs in terms of patient outcomes. For AMI and heart failure, the timely use of proper medicines is crucial for life saving and recovery; however, for pneumonia, C-section, and cholecystectomy, appropriate antibiotic utilization and appropriate surgery are more important. If patients with AMI and heart failure receive medical care that follows the CPs in general but do not receive the proper medications in a timely manner, they may have worse patient status at discharge. With insufficient knowledge about the mechanisms by which CPs work, further studies are necessary to identify the key elements of CPs’ effects on patient clinical outcomes [2,18].

### Limitations

Our study analysed the effects of implementing CPs on medical care at the hospital and case levels as well as the effects on hospital assessments and individual perceptions. However, because CPs are not widely and deeply implemented in hospitals and by physicians and because the analysed objective patient outcomes were limited to the cured, improved or other status at discharge; LOS; and inpatient medical costs, the objective effects of CPs may not be fully demonstrated in our study. Confounding factors, such as the severity of associated comorbidities and patient characteristics, may also affect patient outcomes [19], although we controlled for hospital location and level in the logistic models for the CPs’ objective effects at the hospital level, and we controlled for hospital fixed effects and patient characteristics in the logistic and linear models for the CPs’ objective effects at the case level. Another factor that influenced the demonstration of the CPs’ effects was the quality of the CPs themselves. The national CPs in China, although based on national CGs, may not be applicable in local hospitals, and their adaptation to local hospital CPs may introduce some ineffective services or actions. Readers should be cautious when interpreting the results of CP evaluation studies [24].

## Conclusions

The positive effects of CPs were not highly perceived by the hospitals or by managers and physicians in China. Relatively low involvement in the implementation of and adherence to CPs resulted in no significant effects of CPs on hospital medical care as a whole. However, the chart review for 5 conditions in Chinese hospitals demonstrated that compliance with national CPs reduced LOS and inpatient medical costs. We suggest that CPs be implemented widely and closely followed to improve overall medical care and that further studies be conducted to identify the key elements of CPs that affect patient clinical outcomes.

## Supporting information

S1 Table KPIs for inpatient care for pneumonia (N = 534). (DOCX)

S2 Table KPIs for inpatient care for AMI (N = 487). (DOCX)

S3 Table KPIs for inpatient care for heart failure (N = 426). (DOCX)

S4 Table KPIs for inpatient care for caesarean section (N = 538). (DOCX)

S5 Table KPIs for inpatient care for cholecystectomy (N = 536). (DOCX)
