# Supplementary material for: The perceived and objectively measured effects of clinical pathways' implementation on medical care in China
Source: PLoS One. 2018 May 7;13(5):e0196776. doi: 10.1371/journal.pone.0196776 (PMC5937784; doi:10.1371/journal.pone.0196776)
Supplement: S1 Table — (DOCX) [file pone.0196776.s001.docx]

**S1 Table. KPIs for inpatient care for pneumonia (N=534) ^†^.**

|  |  | **Key process indicators (KPIs)** | **No. of cases** | **Compliance rate (%)** |
| --- | --- | --- | --- | --- |
| 1 | Tests and examinations within 3 days of admission | | 4 | 0.75 |
| 1.1 |  | Routine blood tests | 510 | 95.51 |
| 1.2 |  | Routine urinalysis | 441 | 82.58 |
| 1.3 |  | Routine stool test | 309 | 57.87 |
| 1.4 |  | Hepatorenal function | 491 | 91.95 |
| 1.5 |  | Electrolytes | 485 | 90.82 |
| 1.6 |  | Glycated haemoglobin | 423 | 79.21 |
| 1.7 |  | ESR | 394 | 73.78 |
| 1.8 |  | C-reactive protein | 354 | 66.29 |
| 1.9 |  | Blood gas analysis | 181 | 33.90 |
| 1.10 |  | Infectious disease screening: HBV, HCV, HIV^‡^ | 237 | 44.38 |
| 1.11 |  | Infectious disease screening: RPR^＃^ | 151 | 28.28 |
| 1.12 |  | Pathogenic examination | 281 | 52.62 |
| 1.13 |  | Sputum smear | 207 | 38.76 |
| 1.14 |  | Sputum culture and susceptibility testing | 280 | 52.43 |
| 1.15 |  | Electrocardiogram | 411 | 76.97 |
| 1.16 |  | Chest X-ray | 171 | 32.02 |
| 2 | Patient severity assessed | | 142 | 26.59 |
| 3 | Oxygen saturation test | | 191 | 35.77 |
| 4 | Severe patients (oxygen saturation <92%) received blood gas analysis | | 485 | 90.82 |
| 5 | Timeliness of sputum and blood cultures within 24 hours of admission or before antibiotic utilization | | 243 | 45.51 |
| 6 | Timely and appropriate use of antibiotics within 4-8 hours | | 455 | 85.21 |
| 7 | Reasonable initial antibiotic treatment | | 259 | 48.50 |
| 8 | Appropriate treatment update according to susceptibility testing | | 455 | 85.21 |
| 9 | Assessment of initial treatment protocol within 48-72 hours | | 325 | 60.86 |
| 10 | Appropriate treatment update within 48-72 hours | | 439 | 82.21 |
| 11 | Patient receipt of health education | | 493 | 92.32 |
| 12 | In accordance with discharge standards | | 521 | 97.57 |
| 13 | Appropriate length of stay (7-14 days or deviation for appropriate reasons) | | 505 | 94.57 |

† ICD-10: J13-J15, J18

‡ HBV: Hepatitis B virus, HCV: Hepatitis C virus, HIV: Human immunodeficiency virus

# RPR: Rapid plasma reagin card test
