# Supplementary material for: The perceived and objectively measured effects of clinical pathways' implementation on medical care in China
Source: PLoS One. 2018 May 7;13(5):e0196776. doi: 10.1371/journal.pone.0196776 (PMC5937784; doi:10.1371/journal.pone.0196776)
Supplement: S2 Table — (DOCX) [file pone.0196776.s002.docx]

**S2 Table. KPIs for inpatient care for AMI (N=487) ^†^.**

|  | **Key process indicators (KPIs)** | **No. of cases** | **Compliance rate (%)** |
| --- | --- | --- | --- |
| 1 | Timely use (within 10 minutes after electrocardiogram) of aspirin or clopidogrel at appropriate dose (300 mg) | 280 | 57.49 |
| 2 | Use of β-blocker within 24 hours of admission for patients without contraindications | 316 | 64.89 |
| 3 | Evaluation of left ventricular function within 24 hours of admission | 193 | 39.63 |
| 4 | Reassessment of left ventricular function within one week before discharge | 193 | 39.63 |
| 5 | Reperfusion therapy for STEMI or LBBB patients, but not for NSTEMI and non-LBBB patients^‡^ | 354 | 72.69 |
| 6 | Thrombolytic therapy within 30 minutes of admission for patients with an indication and without contraindications | 426 | 87.47 |
| 7 | PCI within 90 minutes of admission for patients with an indication and without contraindications | 375 | 77.00 |
| 8 | Timely transfer of patients who need PCI but cannot undergo it at the current hospital to another hospital with appropriate resources to perform PCI^＃^ | 461 | 94.66 |
| 9 | Use of aspirin or clopidogrel, β-blocker, ACEI or ARB, and statins during hospitalization for patients with an indication and without contraindications^&^ | 269 | 55.24 |
| 10 | Advice to continue use of aspirin or clopidogrel, β-blocker, ACEI or ARB, and statin after discharge or prescription of them at discharge for patients with an indication and without contraindications | 245 | 50.31 |
| 11 | Patient receipt of health education (no smoking, physical exercise, diet, weight control, and proper treatment of recurrence or worsening) | 477 | 97.95 |
| 12 | Appropriate length of stay (7-14 days or deviation for appropriate reasons) | 436 | 89.53 |

† ICD-10: I21.0-I21.3, I21.4

‡ STEMI: ST elevation myocardial infarction; NSTEMI: non-ST elevation myocardial infarction; LBBB: left bundle branch block

# PCI: Percutaneous coronary intervention

& ACEI: Angiotensin-converting enzyme inhibitor; ARB: Angiotensin II receptor blocker
