# Supplementary material for: The perceived and objectively measured effects of clinical pathways' implementation on medical care in China
Source: PLoS One. 2018 May 7;13(5):e0196776. doi: 10.1371/journal.pone.0196776 (PMC5937784; doi:10.1371/journal.pone.0196776)
Supplement: S3 Table — (DOCX) [file pone.0196776.s003.docx]

**S3 Table. KPIs for inpatient care for heart failure (N=426) ^†^.**

|  | **Key process indicators (KPIs)** | **No. of cases** | **Compliance rate (%)** |
| --- | --- | --- | --- |
| 1 | Assessment of left ventricular function within 24 hours of admission | 177 | 41.55 |
| 2 | Reassessment of left ventricular function within one week before discharge | 173 | 40.61 |
| 3 | Timely use of diuretics and potassium agents within 60 minutes for patients with an indication and without contraindications | 140 | 32.86 |
| 4 | Timely use of ACEI or ARB for patients with an indication and without contraindications^‡^ | 245 | 57.51 |
| 5 | Use of β-blockers only for patients with CHF^#^ | 248 | 58.22 |
| 6 | Use of aldosterone receptor blockers only for patients with severe health failure^&^ | 293 | 68.78 |
| 7 | Advice to continue use of ACEI or ARB, β-blockers, and aldosterone receptor blockers after discharge or prescription of them at discharge for patients with an indication and without contraindications | 208 | 48.83 |
| 8 | Appropriate CRT if necessary^※^ | 417 | 97.89 |
| 9 | Appropriate ICD if necessary^※^ | 418 | 98.12 |
| 10 | Patient receipt of health education or CRT/ICD instruction | 381 | 89.44 |
| 11 | Arrangement of follow-up at discharge | 394 | 92.49 |
| 12 | Appropriate length of stay (7-14 days or deviation for appropriate reasons) | 384 | 90.14 |

† ICD-10: I50.1

‡ ACEI: Angiotensin-converting enzyme inhibitor, ARB: Angiotensin II receptor blocker

# CHF: Chronic heart failure, AHF: Acute heart failure

& Severe heart failure refers to New York Heart Association (NYHA) cardiac function classification (NYHA functional) level III and IV patients

※ CRT: Cardiac resynchronization therapy; ICD: Implantable cardioverter defibrillator
