# Supplementary material for: The perceived and objectively measured effects of clinical pathways' implementation on medical care in China
Source: PLoS One. 2018 May 7;13(5):e0196776. doi: 10.1371/journal.pone.0196776 (PMC5937784; doi:10.1371/journal.pone.0196776)
Supplement: S4 Table — (DOCX) [file pone.0196776.s004.docx]

**S4 Table. KPIs for inpatient care for caesarean section (N=538) ^†^.**

|  | **Key process indicators (KPIs)** | | **No. of cases** | **Compliance rate (%)** |
| --- | --- | --- | --- | --- |
| 1 | Appropriate indication for planned C-section^‡^ | | 537 | 99.81 |
| 2 | Preoperative tests and examinations completed within 2 days of admission | | 232 | 43.12 |
| 2.1 |  | Routine blood tests | 481 | 89.41 |
| 2.2 |  | Routine urinalysis | 380 | 70.63 |
| 2.3 |  | Hepatorenal function (NA when previously received during antenatal care) | 374 | 69.52 |
| 2.4 |  | Coagulation test | 464 | 86.25 |
| 2.5 |  | Electrocardiogram | 349 | 64.87 |
| 2.6 |  | Infectious disease screening: HBV, HCV, HIV, RPR^＃^ | 295 | 54.83 |
| 3 | Prophylactic use of first-generation cephalosporin antibiotics | | 153 | 28.44 |
| 4 | Withdraw of prophylactic antibiotics within 72 hours after delivery | | 414 | 76.95 |
| 5 | Timeliness of surgery (within 2 days of admission) | | 519 | 96.47 |
| 6 | Appropriate anaesthesia (CSEA or CEA) ^&^ | | 333 | 61.90 |
| 7 | Appropriate use of oxytocin during procedure (<20 U) | | 345 | 64.13 |
| 8 | No transfusion or transfusion for appropriate reasons | | 532 | 98.88 |
| 9 | Appropriate postoperative length of stay (≤7 days) | | 518 | 96.28 |
| 10 | Patient receipt of health education prior to discharge | | 478 | 88.85 |
| 11 | Appropriate length of stay (≤9 days or deviation for appropriate reasons) | | 517 | 96.10 |

† ICD-10: the first diagnosis of underlying section of uterus, caesarean operation, ICD-9-CM-3: 74.1 surgical coders

‡ Demand from pregnant women or their relatives, oligohydramnios, foetal factors, chronic foetal distress, cephalopelvic disproportion, complications that affect vaginal delivery, other appropriate indications

# HBV: Hepatitis B virus, HCV: Hepatitis C virus, HIV: Human immunodeficiency virus; RPR: rapid plasma reagin card test

& CSEA: Combined spinal epidural anaesthesia, CEA: Continuous epidural anaesthesia
