# Supplementary material for: The perceived and objectively measured effects of clinical pathways' implementation on medical care in China
Source: PLoS One. 2018 May 7;13(5):e0196776. doi: 10.1371/journal.pone.0196776 (PMC5937784; doi:10.1371/journal.pone.0196776)
Supplement: S5 Table — (DOCX) [file pone.0196776.s005.docx]

**S5 Table. KPIs for inpatient care for cholecystectomy (N=536) ^†^.**

|  | **Key process indicators (KPIs)** | | **No. of cases** | **Compliance rate (%)** |
| --- | --- | --- | --- | --- |
| 1 | Timely tests, examinations and diagnosis (within 2 days) | | 189 | 35.26 |
| 1.1 |  | Routine blood tests | 493 | 91.98 |
| 1.2 |  | Routine urinalysis | 319 | 59.51 |
| 1.3 |  | Hepatorenal function | 496 | 92.54 |
| 1.4 |  | Electrolyte test | 491 | 91.60 |
| 1.5 |  | Coagulation test | 487 | 90.86 |
| 1.6.1 |  | Infectious disease screening: HBV, HCV, HIV^‡^ | 400 | 74.63 |
| 1.6.2 |  | Infectious disease screening: RPR^#^ | 359 | 66.98 |
| 1.7 |  | Abdominal ultrasound | 376 | 70.15 |
| 1.8 |  | Electrocardiogram | 465 | 86.75 |
| 1.9 |  | Chest X-ray | 459 | 85.63 |
| 2 | Severity assessment after admission | | 31 | 5.78 |
| 3 | Consideration of contraindications for cephalosporins and cautious use | | 536 | 100.00 |
| 4 | Performance of antimicrobial susceptibility test | | 218 | 40.67 |
| 5 | Timely use of antibiotics within 4-8 hours | | 283 | 52.80 |
| 6 | Appropriate initial antibiotics | | 279 | 52.05 |
| 7 | Appropriate treatment update according to susceptibility testing | | 492 | 91.79 |
| 8 | Appropriate withdrawal of antibiotics 72-96 hours after return to normal temperature and symptom resolution | | 475 | 88.62 |
| 9 | Timeliness of surgery (within 3 days of admission) | | 413 | 77.05 |
| 10 | Appropriate anaesthesia (GA or CEA) ^&^ | | 535 | 99.81 |
| 11 | No transfusion or transfusion for appropriate reasons | | 531 | 99.07 |
| 12 | Pathological examination | | 518 | 96.64 |
| 13 | Appropriate postoperative length of stay (≤5 days for LC or ≤8 days for OC)^※^ | | 292 | 54.48 |
| 14 | Patient receipt of health education | | 418 | 77.99 |
| 15 | Appropriate length of stay (≤7 days for LC or ≤10 days for OC) | | 229 | 42.72 |

† ICD: K80.0, ICD-9-CM-3: 51.23, 51.24

‡ HBV: Hepatitis B virus, HCV: Hepatitis C virus, HIV: Human immunodeficiency virus

# RPR: Rapid plasma reagin card test

& GA: General anaesthesia, CEA: Continuous epidural anaesthesia

※ LC: Laparoscopic cholecystectomy, OC: Open cholecystectomy
